# Nurses’ bioterrorism preparedness: an evolutionary concept analysis

**DOI:** 10.3389/fpubh.2026.1873477

**Published:** 2026-10-14

**Authors:** Yiwei Luo, Zhengji He, Jiaqing Chen, Yanyan Zhang, Yanhui Liu, Qiushi Zhang, Minye Li, Chen Zhi, Hui Ma

**Affiliations:** 1 People's Liberation Army General Hospital, Beijing, China; 2 School of Nursing, Southern Medical University, Guangzhou, China; 3 Respiratory and Critical Care Medicine, 1st Medical Center of Chinese PLA General Hospital, Beijing, China; 4 School of Nursing, Tianjin University of Traditional Chinese Medicine, Tianjin, China; 5 Joint Logistics Academy, People's Liberation Army National Defence University, Beijing, China

**Keywords:** nurses, bioterrorism preparedness, disaster nursing, public health nursing, conceptual analysis

## Abstract

**Introduction:**

Bioterrorism, involving the deliberate use or release of microorganisms or biologically derived toxins to cause illness or death, poses a distinct threat to public health and healthcare systems. Its intentional nature distinguishes it from naturally occurring infectious disease outbreaks and accidental biological releases. Nurses are essential frontline responders to bioterrorism, yet nurses’ bioterrorism preparedness remains inconsistently defined, limiting the development of targeted education, competency assessment, and response planning. This study aimed to clarify nurses’ bioterrorism preparedness using Rodgers’ evolutionary concept analysis.

**Methods:**

A systematic search of PubMed, Web of Science, CINAHL, MEDLINE, Embase, and ProQuest was conducted from database inception to 8 November 2025. The search identified 537 records, of which 35 sources were included in the concept analysis.

**Results:**

Matrix and thematic analyses identified six defining attributes: knowledge, practical skills, response intention and decision-making, interdepartmental collaboration, operational coordination of equipment and supplies, and continuous learning and iteration. Five antecedent domains were identified: education and training, organisational systems and policies, resource support, prior emergency experience and lessons learned, and threat perception and professional responsibility. The reviewed literature associated preparedness with timely recognition and reporting, protective practices, and coordinated response; descriptions of inadequate preparedness included delays, exposure risks, communication difficulties, and service strain. These associations do not establish causal effects.

**Discussion:**

Nurses’ bioterrorism preparedness is therefore conceptualised as a dynamic, context-sensitive, multidimensional professional competency linking individual capabilities with coordinated organisational response. This conceptualisation may inform subsequent education, assessment, and preparedness research in the context of deliberate biological threats.

## 1 Introduction

Bioterrorism refers to the deliberate use of microorganisms or biologically derived toxins to cause illness or death and remains a serious and evolving threat (1). The 2001 anthrax mail attacks demonstrated the real-world consequences of deliberate biological attacks (2), while advances in biotechnology and synthetic biology have renewed concern about the potential misuse of biological agents (3, 4). Delayed symptom onset, unfamiliar clinical presentations, and potential secondary transmission can hinder early recognition, requiring timely and adaptable healthcare response.

Nurses, as the largest professional group in the healthcare workforce, play a critical frontline role in bioterrorism preparedness and response. Their responsibilities include recognising unusual clinical presentations, identifying potential biological threats, providing direct care to affected individuals, supporting surveillance and early warning, and contributing to infection prevention and risk communication (5). However, previous studies have reported insufficient preparedness among nurses, including gaps in knowledge and training, limited access to exercises and resources, inadequate organisational support, and low confidence in performing specialised response activities (6–12). These persistent gaps make it important to clarify what constitutes bioterrorism preparedness specifically for nurses.

To date, despite the recognized importance of nurses’ bioterrorism preparedness, a consistent nursing-specific definition of this concept remains lacking. Existing literature frequently overlaps with broader concepts such as disaster preparedness, public health emergency preparedness, organisational readiness, and bioterrorism preparedness. National and organisational frameworks generally emphasise system-level hazards, resources, coordination, and response capacity (13, 14), whereas nursing preparedness additionally involves the knowledge, practical skills, willingness, decision-making, and professional responsibilities required of individual nurses in direct care and emergency response (3, 15). Earlier work specifically examining nursing bioterrorism preparedness provided an important foundation for conceptual development (16), yet over the subsequent two decades, changes in the biological-threat environment, experiences arising from emerging infectious diseases, and rapid advances in biotechnology have altered nursing roles and preparedness requirements. These developments underscore the need to re-examine the concept as dynamic and context-dependent rather than as a fixed set of competencies.

This study, therefore, employs Rodgers’ evolutionary concept analysis, which views concepts as dynamic, context-dependent, and subject to change through disciplinary use and broader societal developments (17). This study aimed to clarify the contemporary concept of nurses’ bioterrorism preparedness by identifying its defining attributes, antecedents, consequences, and related concepts while examining how the concept has evolved in response to changing biological-threat contexts.

## 2 Methods

This concept analysis followed Rodgers’ evolutionary concept analysis framework (18), which is particularly appropriate for dynamic, context-dependent concepts such as nurses’ bioterrorism preparedness. Rodgers’ approach is inductive and iterative rather than strictly sequential, with attention to attributes, antecedents, consequences, related terms, and temporal and contextual variation. In this study, these principles guided literature sampling, data extraction, cross-study comparison, and conceptual synthesis.

### 2.1 Search strategy

A systematic literature search was conducted in PubMed, Web of Science, CINAHL, MEDLINE, Embase, and ProQuest from database inception to 8 November 2025. No language filter was applied at the database-search stage. English and Chinese publications were assessed directly; articles in other languages identified during screening were translated using DeepL Translator, where feasible, to support eligibility assessment and extraction of concept-relevant content.

The key search terms used combined three concept blocks with Boolean operators (AND/OR) applied to title, abstract, and keyword fields: (1) Population: “nurse*” OR “nursing staff” OR “nursing student*” OR “nursing manager*,” (2) Content: “Bioterror*” OR “Biological Terrorism” OR “Biological Warfare” OR “Biological Weapon*” OR “Biological Agent*” and (3) Preparedness and response: “preparedness” OR “readiness” OR “education” OR “training” OR “intervention.” Readiness was retained as a complementary retrieval term because some publications use it to describe nurses’ immediate ability or willingness to respond; it was used to improve retrieval sensitivity and was not treated analytically as interchangeable with preparedness. Grey literature was searched via Google Scholar and relevant organisational websites (e.g., WHO, CDC). The database-specific search strategies are provided in Appendix 1.

### 2.2 Inclusion and exclusion criteria

Following Rodgers’ emphasis on concept-relevant literature sampling, studies were eligible when they (1) addressed nurses’ preparedness for bioterrorism, including definitions, characteristics, antecedents, consequences, related terminology, assessment, or educational approaches, and (2) involved registered nurses, nursing students, or nursing managers as the primary population or a clearly identifiable subgroup. Studies of education or training interventions were eligible when they provided relevant conceptual data; an intervention was not required for inclusion.

Exclusion criteria were: (1) studies whose primary focus was general disaster or emergency preparedness with no discernible component or outcome specific to biological agents or bioterrorism; (2) studies centred solely on natural disasters or general disaster preparedness without specific reference to intentional biological threats; and (3) conference abstracts without full text, editorials, commentaries, and other short-form materials lacking sufficient concept-relevant detail. Full-text scholarly dissertations contributing substantive bioterrorism-preparedness data were eligible and were identified as dissertations in Supplementary Table 2.

### 2.3 Study selection and screening

Two researchers independently screened the titles and abstracts of deduplicated records managed in EndNote 20. The same two researchers then assessed potentially eligible full texts against the eligibility criteria. Disagreements were resolved through discussion, with a third reviewer adjudicating any remaining conflicts. Reference lists of included studies were hand-searched for additional relevant articles. The PRISMA flow diagram (Figure 1) was used to report the literature-identification and selection process transparently; the overall study design remained Rodgers’ evolutionary concept analysis.

**Figure 1:**
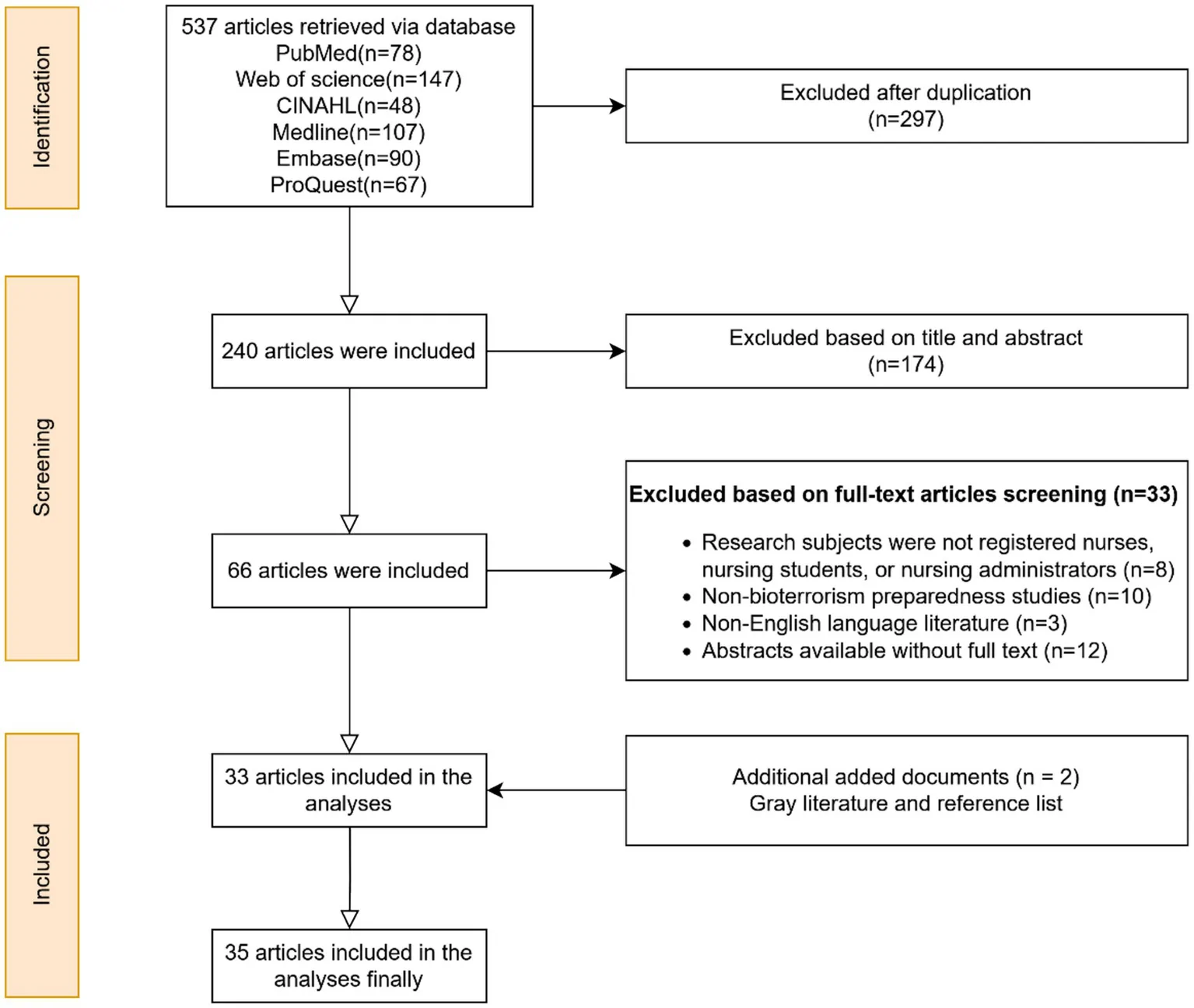
Flow chart of article selection process.

### 2.4 Data extraction

The two researchers independently extracted data using a standardised Excel form, with discrepancies resolved by discussion and final arbitration by a third researcher. In addition to bibliographic details, study design, setting, sample characteristics, and purpose, the form captured concept-relevant data on related concepts, attributes, antecedents, consequences, contextual factors, and assessment instruments.

### 2.5 Data analysis

The same two researchers repeatedly read the included articles and coded text according to Rodgers’ analytic categories, including attributes, antecedents, consequences, related concepts, and contextual features. Matrix and thematic techniques were then used iteratively to identify recurring patterns, with disagreements discussed and, when necessary, adjudicated by a third researcher (19). Particular attention was given to temporal and contextual variation in how preparedness was described across the literature.

Matrix and thematic analyses were used to synthesise the findings (20). First, concept-relevant text was categorised within the extraction matrix. Second, horizontal comparisons across studies and vertical comparisons within each conceptual category were used to consolidate similar content and distinguish recurring themes. Publication period and biological-threat context were considered during these comparisons to support interpretation of the concept’s temporal and contextual evolution (Table 1).

**Table 1:** Operationalisation of Rodgers’ evolutionary concept analysis of nurses’ bioterrorism preparedness.

Steps	Content
Identify the concept and associated expressions	Define “nurses’ bioterrorism preparedness” and its related terms
Determine the setting and scope for data collection	Systematic search across nursing, public health, and multidisciplinary databases without a database language filter; translated assessment was used for other-language records where feasible
Collect data related to attributes, related concepts, contextual factors, antecedents, and consequences	Extract attributes, antecedents, consequences, related concepts, contextual factors, and intervention details
Analyse data to identify characteristics and evolutionary patterns	Iterative thematic and matrix analysis with cross-study comparison of temporal, contextual, and disciplinary variation
Identify implications and hypotheses for further development of the concept	Derive implications for nursing theory, education, training interventions, practice, and research

## 3 Results

### 3.1 Study selection and characteristics

A total of 537 studies were identified. Following duplicate removal and eligibility screening, 35 sources were included in the concept analysis. The selection process is shown in Figure 1. Table 2 summarises the conceptual domains and their cited literature; Supplementary Table 2 describes the publication years, study designs, settings, and assessment tool of the 35 included sources, allowing comparison across time and contexts. In classifying the extracted material, descriptions of what constitutes preparedness were synthesised as attributes; conditions surrounding its development were considered antecedents; and literature-described implications were grouped as consequences. Related concepts were distinguished by differences in scope and use. Among the reviewed publications, earlier work frequently discussed knowledge of biological agents, protective procedures, and willingness to respond (21–26). More recent studies also examined training approaches and factors associated with preparedness (27–29). Read alongside the literature on organisational coordination and learning, these differences suggest a broadening of the concept without implying that every attribute emerged in a fixed chronological sequence. Knowledge and practical skills remained central across contexts.

**Table 2:** Attributes, antecedents and consequences of nurses’ bioterrorism preparedness.

Component type		Domain	Sub-components	Description	References
Antecedents		Education and training	Systematic instruction Simulation platforms Graded exercises with debriefings	Frequently described foundation for knowledge, skills and role clarity	(3, 7, 23, 25, 26, 28, 31, 32)
		Organisational systems and policies	Policy alignment Role delineation Contingency plans	Structural conditions supporting role clarity, coordination and policy-practice integration	(3, 30, 31)
		Resource support	Stockpiles Funding or time Agency guidance	Resources supporting training and operational preparedness	(3, 25, 27, 30)
		Prior emergency experiences and lessons learnt	Past incident lessons Transferable disaster response skills	Experiential reference points informing protocols, training and confidence	(7, 27, 30, 32)
		Threat perception and professional responsibility	Rational risk appraisal Frontline role recognition	Perceptual and professional factors associated with engagement in preparedness	(3, 7, 23, 27, 30–33, 35, 36)
Attributes		Knowledge	Biological agents Response process Infection-control principles	Cognitive framework for recognition and prevention	(3, 7, 21, 23, 30–32)
		Practical skills	PPE procedure Triage and management Communication and logistics	Observable procedural competencies for safe action	(3, 23, 24, 30–33)
		Response intention and decision-making	Agent characteristics Personal safety Training experience Institutional trust	Attitudinal determinants of willingness to act	(3, 7, 30, 31)
		Interdepartmental collaboration	HICS proficiency Multidisciplinary participation Data sharing	Relational capacity for coordinated response	(3, 30, 31)
		Operational coordination of equipment and supplies	Storage requisition knowledge, Surge allocation	Ability to mobilise and manage resources effectively	(30, 32)
		Continuous iteration	Debriefings, lesson integration, Protocol updates	Meta-competency for ongoing learning and improvement	(7, 34)
Consequences	Positive	Early detection	Abnormal case recognition, Specific clinical signatures, Alert activation	Association with more timely identification and reporting	(3, 22, 30)
		Lower exposure and transmission risk	PPE adherence Isolation, Decontamination Public education,	More consistent protective and infection-control practice	(3, 30)
		More coordinated response	Role clarity, Resource mobilisation, Exposure tracing	Clearer roles and coordination within response structures	(3, 5, 30, 31)
		Support for public-health response	Risk communication, Reassurance, Protective guidance	Clearer risk communication and protective guidance	(23, 24, 30, 37–39)
	Negative	Response delays and coordination difficulties	Triage errors, Delayed reporting, Coordination gaps	Delayed recognition/reporting and coordination gaps	(3, 30, 32)
		Greater exposure and transmission risk	Incorrect PPE, delayed isolation, Inadequate decontamination	Potentially greater occupational and patient exposure	(3)
		Communication difficulties and public concern	Poor information management, Mishandled procedures	Unclear or inconsistent risk communication; wider effects untested	(30, 32)
		Service and system strain	Staff refusal, resource depletion, Uncoordinated planning	Workforce reluctance, resource-management difficulties and disrupted service delivery	(3, 30)

^*^HICS, Hospital Incident Command System; CPD, continuing professional development; PPE, personal protective equipment; CBRN, chemical, biological, radiological and nuclear.

### 3.2 Related concepts

No surrogate term was identified in the included literature. Six terms were classified as related concepts because they overlap with nurses’ bioterrorism preparedness but differ in scope, focus, or level of application (Table 3).

1. Nurse emergency preparedness encompasses broad emergency-response knowledge, skills, and attitudes across natural disasters, infectious disease outbreaks, and other emergencies, but does not specifically focus on deliberate biological threats (3, 7, 30).
2. Biological disaster response capability refers primarily to the operational capacity to respond to biological events, including bioterrorism and infectious disease emergencies, and places greater emphasis on response execution than on preparedness as a multidimensional nursing competency (31).
3. Counter-terrorism health preparedness focuses on health-related responses to terrorist threats, including bioterrorism, but has often emphasised willingness to respond rather than the broader combination of knowledge, skills, decision-making, collaboration, and adaptive preparedness (7, 30).
4. Disaster nursing encompasses nursing practice across prevention, preparedness, response, and recovery for both natural and human-made disasters and is therefore broader than bioterrorism-specific preparedness (32, 33).
5. Emergency management refers to an organisational and system-level process encompassing risk reduction, preparedness, response, and recovery (31, 33).
6. Public health emergency preparedness focuses on system-level surveillance, coordination, resource mobilisation, and response across multiple public health threats (30, 34).

**Table 3:** Related concepts.

Category	Term	Key characteristics	Distinction from core concept
Related concept	Nurse emergency preparedness (3, 7, 30)	Broad emergency knowledge, skills, attitudes	Includes non-intentional events
	Biological disaster response capability	Operational response to biological events	Focuses on execution rather than preparation
	Counter-terrorism health preparedness (7, 30)	Willingness to respond to terrorist threats	Limited to individual attitudinal factors
Related concept	Disaster nursing (32, 33)	Care across all disaster phases	Encompasses natural and man-made disasters
	Emergency management (33)	Organisational planning and phases	System-level rather than nursing-specific
	Public health emergency preparedness (34)	Surveillance and coordination	Broader public health system focus

### 3.3 Attributes

Attributes are essential characteristics that define a concept (18). Six core attribute domains were identified: knowledge, practical skills, response intention and decision-making, interdepartmental collaboration, operational coordination of equipment and supplies, and mechanisms for continuous iteration. Although several of these attributes also appear in broader disease or infectious disaster preparedness, they were considered components of nurses’ bioterrorism preparedness when the literature linked them to the recognition, protection, reporting, coordination, or response requirements associated with deliberate biological threats.

#### 3.3.1 Knowledge

Knowledge provides the cognitive foundation of nurses’ bioterrorism preparedness and includes understanding of priority biological agents, their clinical manifestations and transmission routes, recognition of unusual clinical or epidemiological patterns suggestive of deliberate release, and relevant response procedures (3, 7, 21–23, 30–32). It also encompasses knowledge of reporting pathways, isolation, and infection-control principles, and treatment or prophylactic measures applicable when a bioterrorism event is suspected or confirmed.

#### 3.3.2 Practical skills

Practical skills refer to nurses’ ability to translate bioterrorism-related knowledge into safe and effective action. Core elements include appropriate use of personal protective equipment, decontamination, triage and patient management, surveillance documentation, communication, and implementation of infection-control procedures (3, 22–24, 27, 30–33). In a bioterrorism context, these skills must be applied while simultaneously considering clinical urgency, transmissibility, exposure risk, and resource constraints.

#### 3.3.3 Response intentions and decision-making

Response intention and decision-making describe nurses’ willingness and capacity to act under conditions of uncertainty, deliberate harm, and occupational risk. Willingness to respond is influenced by perceived threat severity, personal and family safety concerns, previous training, and confidence in organisational protective measures (3, 7, 30, 31). This attribute, therefore, extends beyond knowledge and technical competence to include risk appraisal, professional commitment, and timely decision-making during a suspected bioterrorism event.

#### 3.3.4 Interdepartmental collaboration

Interdepartmental collaboration refers to nurses’ capacity to operate within multidisciplinary and interagency response structures. It includes role clarity, communication within command systems, and timely information sharing with relevant clinical departments, public-health agencies, laboratories, and emergency services during a bioterrorism response (3, 30, 31).

#### 3.3.5 Operational coordination of equipment and supplies

Operational coordination of equipment and supplies focuses on the nurse’s ability to mobilise, prioritise, and appropriately use essential resources during a bioterrorism response. Unlike the mere availability of resources, this attribute reflects the capacity to coordinate equipment, protective supplies, medications, and other resources under surge conditions associated with deliberate biological threats (30, 32).

#### 3.3.6 Continuous iteration mechanism

Continuous learning and iteration represent the dynamic and adaptive nature of bioterrorism preparedness. It involves learning from exercises and actual events, identifying deficiencies through debriefing and evaluation, updating protocols and contingency plans, and incorporating new evidence and threat information into continuing education and preparedness activities (7, 34). Unlike access to education and training, which is an antecedent, this attribute concerns nurses’ capacity to incorporate lessons into subsequent preparedness activities.

### 3.4 Antecedents

Antecedents are conditions or events that precede or are associated with the development of a concept (17). Five antecedents of nurses’ bioterrorism preparedness were identified: education and training, organisational systems and policies, resource support, prior emergency experiences and lessons learned, and nurses’ threat perception and professional responsibility.

#### 3.4.1 Education and training

Education and training constitute a primary antecedent of nurses’ bioterrorism preparedness (7, 25–28, 32). Relevant preparation includes pre-registration and continuing professional education addressing biological threats, recognition and response procedures, infection control, protective measures, triage, communication, and role responsibilities. Simulation and emergency exercises further provide opportunities to apply knowledge, clarify roles, identify deficiencies, and strengthen response competence (3, 7, 23, 25–28, 31, 32).

#### 3.4.2 Organisational systems and policies

Organisational systems and policies provide the structural conditions necessary for nurses to develop and apply bioterrorism preparedness. These include clear institutional plans, defined nursing roles, reporting and command pathways, integration with national or local biological-threat response requirements, and regular testing and revision of preparedness procedures (3, 22, 27, 30, 31).

#### 3.4.3 Resource support

Resource support refers to the material, financial, technical, and organisational resources required to sustain bioterrorism preparedness. Adequate protective equipment, isolation capacity, medications, training resources, protected training time, and access to technical guidance facilitate both preparedness development and effective response (3, 22, 25, 27, 30).

#### 3.4.4 Prior emergency experiences and lessons learned

Experiences from previous bioterrorism events and relevant infectious disease emergencies can inform subsequent preparedness, although these contexts are not conceptually equivalent. Direct lessons from events, such as the 2001 anthrax attacks, have informed recognition, reporting, protective measures, and response planning (22, 32). Naturally occurring outbreaks, including SARS and COVID-19, may provide transferable lessons in infection control, surge management, communication, resource allocation, and teamwork, but are not themselves classified as bioterrorism events because deliberate intent is absent (7, 22, 27, 30, 32).

#### 3.4.5 Threat perception and professional responsibility

Perceiving bioterrorism as a credible occupational and public-health threat, together with recognising professional responsibility during such events, can motivate nurses to engage in preparedness activities (3, 7, 22, 23, 27, 30–33, 35, 36). Appropriate risk appraisal and awareness of frontline responsibilities may therefore facilitate knowledge acquisition, skill development, participation in training, and willingness to respond.

### 3.5 Consequences

Consequences are outcomes described in the literature as occurring in relation to the presence or absence of a concept. In this analysis, they are treated as literature-reported associations and conceptual implications rather than causal effects established by the present study. Four positive and four adverse consequences were identified.

#### 3.5.1 Positive outcomes

1. Early detection of biological events.

Across the reviewed literature, greater preparedness was associated with more timely recognition of unusual clinical or epidemiological patterns suggestive of deliberate biological-agent release and earlier initiation of reporting and response procedures (3, 22, 27, 30).

1. Reduced exposure and transmission risk

Preparedness was associated with more consistent application of infection-control, isolation, protective, and decontamination measures, with potential implications for reducing occupational and patient exposure and secondary transmission risk (3, 22, 27, 30).

1. More coordinated emergency response

Prepared nurses were described as better able to work within coordinated response structures, perform defined roles, and support timely triage, communication, and resource mobilisation during bioterrorism events (3, 5, 30, 31).

1. Support for public-health response

Preparedness was linked in the literature to clearer risk communication, appropriate protective behaviours, and more coordinated healthcare responses, while wider effects on public confidence and public-health protection require empirical investigation (23, 24, 30, 37–39).

#### 3.5.2 Negative consequences

1. Response delays and coordination difficulties

Insufficient preparedness was associated with delayed recognition and reporting, inappropriate triage, unclear role performance, and coordination difficulties that may reduce opportunities for timely containment (3, 22, 30, 32).

1. Greater exposure and transmission risk

Deficiencies in protective practices, isolation, or decontamination were described as increasing the risk of occupational and patient exposure and secondary transmission following a deliberate biological-agent release (3, 22).

1. Communication difficulties and public concern

Inadequate or inconsistent communication during a suspected bioterrorism event was linked in the literature to unclear risk information and public concern (22, 30, 32). This analysis cannot determine effects on population-level anxiety or confidence.

1. Service and system strain

Poor preparedness was associated with workforce reluctance, resource-management difficulties, and disrupted service delivery, indicating potential strain on the healthcare system’s capacity to sustain an effective bioterrorism response (3, 22, 30).

### 3.6 Proposed conceptual definition

Nurses’ bioterrorism preparedness is an evolving, context-sensitive professional competency shaped by emerging intentional biological threats, global health security imperatives, and interdisciplinary emergency response developments. It is characterised by specialised knowledge of biological agents and response protocols; procedural skills for protection, infection control, triage, and response; willingness and decision-making under conditions of deliberate threat and uncertainty; collaborative capacity within multidisciplinary and interagency response systems; effective coordination of essential resources; and continuous learning and adaptation through exercises, evaluation, and lessons from relevant events. Nurses’ bioterrorism preparedness therefore represents not merely possession of knowledge or technical skills, but the integrated capacity to recognise and respond to suspected or confirmed deliberate biological threats within a changing healthcare and security environment.

## 4 Discussion

This study clarifies the concept of nurses’ bioterrorism preparedness, delineating its attributes, antecedents, consequences, and distinguishing it from related concepts. This approach addresses the conceptual gap in nursing practice and research concerning bioterrorism preparedness.

Compared with seminal work by Rebmann (16), which used Walker and Avant’s concept analysis framework in the context of the September 11 attacks and the 2001 anthrax incidents, the present study examines its temporal and contextual variation using Rodgers’ evolutionary approach. Rebmann’s analysis established nurses’ bioterrorism preparedness as a continual process of becoming better equipped, with particular emphasis on knowledge and operational readiness. Consistent with that earlier work, the present study also identifies knowledge as a core attribute, while the reviewed literature also emphasises response intention and decision-making, interdepartmental collaboration, operational coordination of equipment and supplies, and continuous iteration mechanisms. These findings suggest a broader conceptual emphasis on organisational and adaptive dimensions alongside individual preparedness. In addition, the reviewed literature describes preparedness as a dynamic process informed by exercises, debriefing, lessons learned, and protocol revision. This interpretation is consistent with Rodgers’ view that concepts are context-dependent and may change through disciplinary and organisational use.

Across the included publications, earlier discussions often centred on biological-agent knowledge, protective procedures, and willingness to respond, while later work also addressed simulation, organisational support, multidisciplinary coordination, and learning from exercises and events. These emphases overlap across periods; they indicate contextual broadening in the published discussion of the concept rather than a uniform sequence in which individual attributes appeared. Recent research on clinical nurses’ preparedness (29) and a broader synthesis of disaster-preparedness domains (40) provide further context for considering individual and organisational dimensions together, although neither establishes the effectiveness of a specific bioterrorism intervention. These sources also differ in scope: bioterrorism-specific studies consider the demands associated with intentional biological threats, whereas broader disaster-preparedness research addresses organisational domains shared by multiple emergency types. We therefore use the latter for contextual comparison, not as evidence that the concepts are interchangeable. Clinical and organisational tasks may also overlap with those discussed in naturally occurring infectious disease emergencies (45), but recognition and reporting in the context of a suspected deliberate release remain part of the focal concept. This distinction helps explain why nurses’ bioterrorism preparedness cannot be inferred from general emergency knowledge or technical competence alone.

As frontline responders, nurses are expected to recognise and report suspected biological threats and follow appropriate protective procedures (29). In the reviewed literature, early recognition and reporting, protective practice, and coordinated response are described as relevant consequences or expressions of preparedness; this concept analysis does not establish their causal effects on transmission or casualties. For practice, preparedness assessments and structured debriefing may help identify gaps across the proposed attributes, although their effects on response outcomes require empirical evaluation (40).

Gaps in bioterrorism-related nursing education, knowledge, and skills have been reported (29). The identified attributes provide a conceptual basis for considering biological-agent knowledge, simulation, role clarification, and interdisciplinary exercises in undergraduate and continuing education (41). Educational and assessment approaches could also address response intention, decision-making, and threat perception; their effects on clinical outcomes would require separate evaluation. For research, measurement tools could be developed and validated to represent the six proposed attributes of preparedness. Longitudinal and cross-cultural studies could examine how the expression of these attributes varies across threat contexts and settings.

Organisational systems and policies were identified as antecedent conditions relevant to nurses’ bioterrorism preparedness. National frameworks provide the context for training, reporting pathways, and resource planning. At the organisational level, protective equipment, training time, clear nursing roles, and response protocols are among the conditions discussed in the literature (42). The 2001 anthrax attacks provided an important context for subsequent preparedness discussions and policy development (43). As the biological-threat context changes, the meaning and practical expression of preparedness may also require periodic re-examination (43). Reviewing gaps between policy and nursing practice may help inform future conceptual and empirical work. These considerations are consistent with Rodgers’ context-sensitive view of concept development.

### 4.1 Limitations

This analysis offers a context-dependent account of nurses’ bioterrorism preparedness rather than a final definition; it may require revision as evidence and biological-threat contexts evolve. In addition, the terminology used in the literature on bioterrorism preparedness is heterogeneous, with related terms such as biological emergency preparedness, biological incident preparedness, disaster preparedness, and emergency readiness sometimes used inconsistently or interchangeably. This may have affected the identification and inclusion of relevant studies and may also have introduced some uncertainty in defining the conceptual boundaries of nurses’ bioterrorism preparedness. Although no database language filter was applied, English and Chinese texts were assessed directly while other-language records relied on machine-assisted translation where feasible; nuances relevant to conceptual interpretation may therefore have been lost or altered. Figure 1 reports three non-English full-text exclusions; the original record-level reasons for these decisions could not be reconstructed, which may have narrowed the conceptual evidence base.

Rodgers’ evolutionary concept analysis encourages researchers to gather data and evidence from multiple disciplines to develop a comprehensive understanding of concepts (44). Although a structured literature search was used to support transparent sampling, this study was conducted as an evolutionary concept analysis rather than a systematic review of effectiveness or prevalence. The majority of the content is derived from the field of nursing, which may lead to an incomplete understanding of the concept. Perspectives from related disciplines, such as public health, emergency management, epidemiology, and security studies, may therefore be underrepresented. Furthermore, Rodgers’ evolutionary concept analysis is inherently interpretive, and the identification and categorisation of attributes, antecedents, consequences, and related concepts may be influenced by researchers’ judgement. Although the analysis followed a structured process, alternative interpretations of the literature remain possible. The included corpus contains a dissertation and a related journal article by the same author; overlapping conceptual content should not be treated as independent corroboration.

## 5 Conclusion

This study conceptualises nurses’ bioterrorism preparedness as a dynamic, context-sensitive professional competency characterised by knowledge, practical skills, response intention and decision-making, interdepartmental collaboration, operational coordination of equipment and supplies, and continuous learning and iteration. The identified antecedents indicate that this competency is situated within educational, organisational, resource, experiential, and professional contexts, while the consequences identified in the literature describe situations through which preparedness or its absence may become apparent. The analysis also suggests that the concept has broadened from a predominantly individual emphasis toward a more system-integrated and adaptive understanding. These findings provide a conceptual basis for future measurement, education, and empirical research; they do not establish causal effects of individual preparedness components on clinical or system outcomes. As an evolutionary concept, nurses’ bioterrorism preparedness should continue to be refined as biological threats, healthcare systems, and nursing roles change.

## Data Availability

The original contributions presented in the study are included in the article/Supplementary material; further inquiries can be directed to the corresponding author.

## Supplementary material

Supplementary Appendix 1 Database-specific search strategies.

Supplementary Table 2 Characteristics of the 35 sources included in the concept analysis.
