# Supplementary material for: Nurses’ bioterrorism preparedness: an evolutionary concept analysis
Source: Front Public Health. 2026 Oct 14;14:1873477. doi: 10.3389/fpubh.2026.1873477 (PMC13729762; doi:10.3389/fpubh.2026.1873477)
Supplement: Supplementary file 1 [file Supplementary_file_1.pdf]

Appendix 1 Database search strategies

Search period: database inception to 8 November 2025. No language filter was applied at the database-search stage. English and Chinese publications were assessed directly; publications in other languages were translated using DeepL Translator, where feasible, for eligibility assessment and concept-relevant data extraction.

The search strategy combines three concept blocks: population, biological threats, and preparedness and response. Readiness is a complementary retrieval term and is not treated as analytically interchangeable with preparedness. Grey literature was searched using Google Scholar and relevant organisational websites, including WHO and CDC.

PubMed

Records identified as reported in Figure 1: 78.

```
(nurse*[tiab] OR "nursing staff"[tiab] OR "nursing student*"[tiab] OR "nursing manager*"[tiab])
AND
(bioterror*[tiab] OR "biological terrorism"[tiab] OR "biological warfare"[tiab] OR "biological
weapon*"[tiab] OR "biological agent*"[tiab])
AND
(preparedness[tiab] OR readiness[tiab] OR education[tiab] OR training[tiab] OR
intervention[tiab])
```

Web of Science

Records identified as reported in Figure 1: 147.

```
TS=(
(nurse* OR "nursing staff" OR "nursing student*" OR "nursing manager*")
AND
(bioterror* OR "biological terrorism" OR "biological warfare" OR "biological weapon*" OR
"biological agent*")
AND
(preparedness OR readiness OR education OR training OR intervention)
)
```

CINAHL via EBSCOhost

Records identified as reported in Figure 1: 48.

```
(TI nurse* OR AB nurse* OR TI "nursing staff" OR AB "nursing staff" OR TI "nursing student*" OR AB "nursing student*" OR TI "nursing manager*" OR AB "nursing manager*")
AND
(TI bioterror* OR AB bioterror* OR TI "biological terrorism" OR AB "biological terrorism" OR TI "biological warfare" OR AB "biological warfare" OR TI "biological weapon*" OR AB "biological weapon*" OR TI "biological agent*" OR AB "biological agent*")
AND
(TI preparedness OR AB preparedness OR TI readiness OR AB readiness OR TI education OR AB education OR TI training OR AB training OR TI intervention OR AB intervention)
```

MEDLINE via Ovid

Records identified as reported in Figure 1: 107.

```
1. (nurse* OR "nursing staff" OR "nursing student*" OR "nursing manager*").ti,ab,kf.
2. (bioterror* OR "biological terrorism" OR "biological warfare" OR "biological weapon*" OR "biological agent*").ti,ab,kf.
3. (preparedness OR readiness OR education OR training OR intervention).ti,ab,kf.
4. 1 and 2 and 3
```

Embase

Records identified as reported in Figure 1: 90.

```
('nurse*':ti,ab,kw OR 'nursing staff':ti,ab,kw OR 'nursing student*':ti,ab,kw OR 'nursing
manager*':ti,ab,kw)
AND
('bioterror*':ti,ab,kw OR 'biological terrorism':ti,ab,kw OR 'biological warfare':ti,ab,kw OR
'biological weapon*':ti,ab,kw OR 'biological agent*':ti,ab,kw)
AND
('preparedness':ti,ab,kw OR 'readiness':ti,ab,kw OR 'education':ti,ab,kw OR 'training':ti,ab,kw
OR 'intervention':ti,ab,kw)
```

ProQuest

Records identified as reported in Figure 1: 67.

```
TI,AB,SU(nurse* OR "nursing staff" OR "nursing student*" OR "nursing manager*")
AND
TI,AB,SU(bioterror* OR "biological terrorism" OR "biological warfare" OR "biological weapon*"
OR "biological agent*")
AND
TI,AB,SU(preparedness OR readiness OR education OR training OR intervention)
```

Total database records: 78 + 147 + 48 + 107 + 90 + 67 = 537 records, consistent with Figure 1. No additional language or publication-date filter is specified in these strings beyond the search cutoff reported above.
